# Bone Substitute Effect on Vascularization and Bone Remodeling after Application of phVEGF_165_ Transfected BMSC

**DOI:** 10.3390/jfb3020313

**Published:** 2012-04-19

**Authors:** Florian Geiger, Mirjam Beverungen, Helga Lorenz, Julia Wieland, Michael Fehr, Philip Kasten

**Affiliations:** 1 Division of Experimental Orthopedics, Orthopedic University Hospital of Heidelberg, Heidelberg 69118, Germany; Email: mirjam.beverungen@gmx.de (M.B.); Helga.lorenz@ortho.uni-hd.de (H.L.); jwieland@harlan.com (J.W.); philip.kasten@uniklinikum-dresden.de (P.K.); 2 Department of Spine Surgery, Orthopedic University Hospital Friedrichsheim, J.W. Goethe University of Frankfurt, Frankfurt, Main 60528, Germany; 3 University of Veterinary Medicine Hannover, Hannover 30559, Germany; Email: michael.fehr@tiho-hannover.de; 4 Harlan Cytotest Cell Research GmbH, Rossdorf 64380, Germany; 5 Department of Orthopedic Surgery, University of Dresden, Dresden 01307, Germany

**Keywords:** angiogenesis, bone substitutes, VEGF, osteogenesis, BMSC

## Abstract

VEGF (vascular endothelial growth factor) promotes vascularization and remodeling of bone substitutes. The aim of this study was to examine the effect of distinct resorbable ceramic carriers on bone forming capacities of VEGF transfected bone marrow stromal cells (BMSC). A critical size defect of the radius in rabbits was filled either by a low surface scaffold called beta-TCP (tricalciumphsphate) or the high surface scaffold CDHA (calcium deficient hydroxy-apatite) loaded with autologous BMSC, which were either transfected with a control plasmid or a plasmid coding for phVEGF_165_. They were compared to unloaded scaffolds. Thus, six treatment groups (n = 6 in each group) were followed by X-ray over 16 weeks. After probe retrieval, the volume of new bone was measured by micro-CT scans and vascularization was assessed in histology. While only minor bone formation was found in both carriers when implanted alone, BMSC led to increased osteogenesis in both carriers. VEGF promoted vascularization of the scaffolds significantly in contrast to BMSC alone. Bone formation was increased in the beta-TCP group, whereas it was inhibited in the CDHA group that showed faster scaffold degradation. The results indicate that the interaction of VEGF transfected BMSC with resorbable ceramic carrier influences the ability to promote bone healing.

## 1. Introduction

Large bone defects are still a challenge in orthopedic surgery and require the use of distraction osteogenesis or the application of bone grafts or bone substitutes. The bridging of large defects with stable bone substitutes is, however, limited by its vascularization, as angiogenesis must precede osteogenesis in skeletogenesis as well as in fracture healing [1]. Bone grafts become atrophic sequesters if they exceed a critical size and are not vascularized sufficiently [2,3]. 

In order to stimulate all three components of bone healing, we combined a stable and osteoconductive scaffold with osteogenic cells and a growth factor (GF). The GF we used in the current study was VEGF (vascular endothelial growth factor) as we figured angiogenesis and vascularization to be essential for osteogenesis. 

During endochondral bone formation, VEGF couples blood vessel formation, chondrocyte apoptosis, cartilage remodeling and endochondral ossification at the growth plates and thus plays a crucial role in bone remodeling and regeneration [4]. Although VEGF was first used to treat vascular diseases [5], its role in skeletogenesis and bone healing soon became obvious. In the initial phase of bone healing, VEGF is expressed by angioblasts, osteoprogenitor cells, chondrocytes and osteoblasts [6]. Peng *et al*. [7] and other authors [8,9] found a synergistic enhancement of bone formation after application of muscle-derived stem cells which expressed either BMP-4, VEGF or both growth factors.

In a previous study, it was demonstrated that vascularization of otherwise atrophic non-unions is feasible by applying plasmid-DNA encoded for phVEGF_165_ on a collagen scaffold, which then acted as a “gene activated matrix” (GAM) [10]. This model was transferred to a more stable bone substitute, *i.e.*, a coralline scaffold [11]. Although a direct gene transfer via a GAM obviously worked well on a collagen matrix [10], it did not in more stable scaffolds, such as the coralline scaffold [11]. Consequently, we moved on to use transfected bone marrow stromal cells (BMSCs) to evaluate the influence of VEGF, as we knew from former experiments that transfected BMSCs maintain a steady level of VEGF at the site of the bony defect. Indeed, phVEGF165 transfected BMSCs led to a higher vascularization and faster resorption of the bone substitute [11]. However, the coralline carrier was resorbed too fast, and osteogenesis decreased in contrast to administration of BMSCs on the scaffold alone. Therefore, the aim of the present study was to identify a more suitable scaffold for bone regeneration by phVEGF_165_ transfected BMSCs. 

Therefore, two synthetic ceramic scaffolds that are resorbed slower than the coralline one were used: comparisons were made [12] between β-tricalciumphosphate (β-TCP), which has been widely used in bone regeneration, and a novel bone substitute called calcium-deficient hydroxyapatite (CDHA). CDHA is a member of the high-specific surface area (SSA) ceramic group, with a SSA of 20–80 m^2^/g, approaching the values of about 80 m^2^/g found in natural bone [13,14]. According to the literature [13,14], cells adhere more easily to high-SSA ceramics, e.g., CDHA, than to low-SSA scaffolds like β-TCP. In order to balance the need for a high porosity to enable cell migration and the need for stability, the porosity of the synthetic ceramics was adjusted to be 75 vol% for the β-TCP and 85 vol% for the CDHA. 

In order to eliminate a side effect of the transfection process, control BMSC underwent a transfection procedure with a plasmid that did not carry the VEGF gene.

The unloaded scaffolds served as additional control. As the animal model was verified before by our group [10,11] as well as by other authors [15,16,17], we did not repeat the trial with empty critical size defects. 

In summary, we compared the effect of BMSCs transfected with phVEGF_165_ on two different scaffolds. The hypothesis was that BMSCs transfected with phVEGF165 lead to different vascularization, bone turnover and finally bone formation in the two bone substitutes due to different material properties such as porosity, surface area and resorption kinetics of the β-TCP and CDHA scaffolds. 

## 2. Experimental Section

### 2.1. Ceramic Scaffolds

CDHA and β-TCP ceramic cylinders of 15 mm length and 4 mm diameter [Robert Mathys Foundation (RMS), Switzerland] were produced in an emulsion process as described earlier [18,19]. β-TCP has an overall porosity of 75 vol% with an average macroporosity (pores Ø 0.2–0.6 mm) of 55 vol% and a microporosity (pores Ø <5 µm) of 23 vol%. The specific surface area of β-TCP is below 0.5 m^2^/g. In contrast, CDHA has a specific surface area that approximates to 48 m^2^/g, while its total porosity is similar (total porosity 85 vol%, with 54% macropores and 31% micropores).

### 2.2. Bone Marrow Aspiration and BMSC Expansion

Bone marrow was aspirated and prepared from the tibia of all rabbits about 18 days before surgery as described previously [10,11]. The bone marrow was digested, cultured and filtered according to a standard protocol [20]. When reaching confluence, the cells were harvested by trypsination and cultured at a density of 3,000 to 6,000/cm^2^ until passage 4 to 6 equaling 18–21 population doublings.

### 2.3. Plasmid Preparation and Transfection of Rabbit Bone Marrow Stromal Cells (BMSC)

Human VEGF (GeneBank Acc. No. XM 004512; Bethesda, MD, USA) was integrated into the pCR3.1 plasmid (Invitrogen, Karlsruhe, Germany) as published previously [5,10].

Transfection was performed with 1 µg of plasmid DNA of hVEGF_165_ or empty pCR3.1 combined with 3 µL of metafectene per 100,000 cells in serum free medium. After 6 hours, the medium was changed to BMSC culture medium and cells were kept overnight. 5 × 10^6^ of the transfected cells were then seeded on each scaffold [10,11]. 

### 2.4. Loading of Scaffolds with Cells

All scaffolds were first incubated in 40 µg/mL fibronectin (human fibronetin F-2006, Sigma) in PBS at 4 °C overnight for coating with fibronectin. This was also done for the scaffolds which were not used for seeding of BMSCs. The next day, the matrix was incubated with 3 mL BMSCs suspension, containing 5 × 10^6^ cells on a roller for 1.5 hours. Then, the scaffolds were put into a sterile 6-well plate and the cell suspension was centrifuged at 470× g for 5 min to recover the remaining BMSCs. The cell pellet was resuspended in 70 µL culture medium and pipetted onto the scaffolds immediately before transplantation [11]. A seeding efficacy of more than 85% for both scaffolds was measured with this technique [14].

### 2.5. Surgical Procedure

An established non-union protocol, which was adapted from Wittbjer *et al*., was used which represents a bone gap of a critical size [16], like it would appear after resection of a pseudarthrosis or tumor or after a comminuted fracture of a long bone. This standard non-union model was verified by our group and others before [10,11,15]. We proved that all animals with the unfilled gap developed an atrophic non-union. Therefore this was not repeated.

Briefly, unilateral 15 mm long critical size defects were prepared in the radial diaphysis of New Zealand white rabbits (NZWR) and the ceramics were implanted press-fit. Due to the synostosis of ulna and radius no further stabilization is needed in rabbits [10,11]. Although a certain micromotion of the implant might be possible due to resorption of the scaffold, no dislocation could be found in the subsequent control radiographs. After 16 weeks the animals were euthanized. Samples were excised *en bloc* with the surrounding soft tissues, and immediately placed in paraformaldehyde 4%.

Animals were treated in compliance with the guiding principles in the “care and use of animals”. The “committee on animal experimentation of Baden-Wuerttemberg (Germany)” approved the experiment. Six- to nine-month old skeletally mature female New Zealand White Rabbits (NZWR) (n = 36) weighing 3.6–5.0 kg (mean 4.5 kg) were kept in separate cages, fed a standard diet and allowed free mobilization during the study. The rabbits were anesthetized as described previously [10]. Postoperatively, four mg/kg carprofen were administered as needed for pain. There were no differences in carprofen administration between the groups. Water and food were supplied *ad libitum*. 

The 36 rabbits were randomly assigned to 6 treatment groups. Each group consisted of 6 animals. Skeletal maturity was proven by X-ray.

### 2.6. Radiographic Evaluation

Standardized anterior-posterior and lateral radiographs were taken immediately postoperatively and every four weeks thereafter until sacrifice after week 16. Radiographs helped to assess proper placement of the bone substitute and progress of bone healing. The degree of bony healing and bridging of the defect was measured semi-quantitatively as described previously [10]. Bony bridging of the gap of up to 25% was declared as “minor bone formation”, between 25 and 75% as “major bone formation” and a bridging from one side to the other as “full healing”. The number of animals with a certain percentage of bone healing is given in table 1. 

As standard X-rays do not allow the volume of new bone to be measured, a µ-CT was performed after sacrificing the animals.

### 2.7. Micro-Computer Tomography (µCT)

µ-CT was performed according to a standard protocol [10,11] after retrieval of the samples in week 16. Each specimen containing the 1.5-cm segmental defect and 0.5 cm of proximal and 0.5 cm of distal cortical bone adjacent to the defect was examined with a micro-computer tomography (µ-CT) system (Fanbeam Micro-CT; Stratec, Stuttgart, Germany). The microfocus of the X-ray source of the µ-CT system had a spot size of 7 µm and a maximum voltage of 36 kV. The image matrix was 1,024 × 1,024 pixels. The specimens were placed in a sample holder filled with water. They were oriented in such a way that the long axis of the block was parallel to the axis of the sample holder. A high-resolution protocol (slice thickness 120 µm, feed 60 µm, pixel size 60 µm) was applied. Depending on the length of the specimens, up to 180 slices were scanned perpendicular to the block. 

To determine the amount of newly-formed bone tissue, the best threshold for the CDHA and β-TCP scaffold and rabbit bone, standard phantoms of each scaffold and several bones were scanned prior to our investigation for calibration.

Furthermore, corresponding histologic and µ-CT slices were compared to check the proper discrimination between bone and scaffolds.

The percentage of newly-formed bone was calculated by dividing the number of voxels containing newly-formed bone by the total number of voxels within a virtual tube corresponding to the bone defect using imaging analysis software (VGStudio Max 1.2.1; Volume Graphics, Heidelberg, Germany). Only bone within this virtual bone was measured, thus bone outside of the scaffold was not considered. 

To determine the degradation of the scaffold after 16 weeks, the volume of the remaining scaffold was given as percentage to the initial scaffold volume.

### 2.8. Histology and Immunohistochemical Staining for Blood Vessels

**Figure 1:**
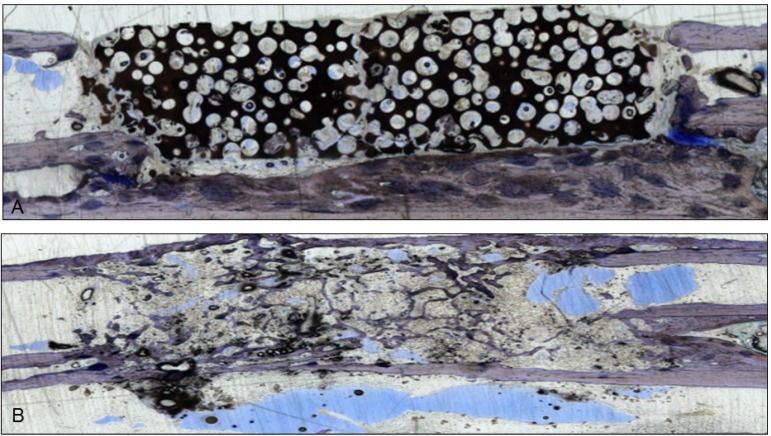
Two sections stained with giemsa and toluidine-blue show the integration of the scaffolds in the bone gap. They were used to verify the µ-CT scans. (**A**) This section represents an example of CDHA without VEGF. The scaffold is still visible and barely degenerated, but bone grew into the lacunae;(**B**) After administration of VEGF-transfected BMSCs, almost the whole CDHA-scaffold had degraded and new bone filled the gap. Endothelial cells were stained with a monoclonal mouse anti-human CD31 antibody (Clone: JC70A Dako Cytomation Glastrup, Denmark; dilution 1:20) according to the manufacturer’s instructions [10,11]. All sections were examined with a light microscope by two independent observers.

**Figure 2:**
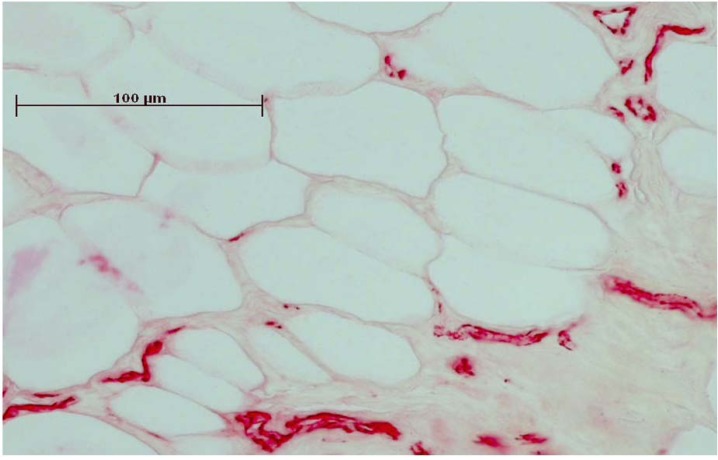
Immunhistochemical staining of CD31 made the endothelial cells of the vessels clearly visible. Before staining, the sections were embedded in Technovit and ground to 50 µm thickness.

Staining procedures were performed using standard protocols [10,11,21]. Retrievals were embedded in Technovit (Technovit 9100 N, Heraeus-Kulzer, Wehrheim, Germany) and ground into sections of about 50 µm thickness (Exakt Apparatebau, Hamburg, Germany). As described previously, these sections were deacrylated and prepared for staining [11]. One section from each sample was stained with giemsa and toluidine-blue (Figure 1; Waldeck GmbH&Co Division Chroma, Münster, Germany) and three were used for immune-histological staining of the vessels (Figure 2).

### 2.9. Histomorphometric Evaluation

On each slide, six standardized regions of interest (ROI) with an area of 0.6 mm² were photographed at a 10× magnification with the Axioplan 2 (Carl-Zeiss, Oberkochen, Germany) [10,11]. Slices were stained by a blinded investigator, and blood vessels were marked by two independent examiners blinded for groups and treatment. The number of vessels in each ROI was determined with the help of the Axiovision 4.4 program (Carl-Zeiss, Oberkochen, Germany). 

### 2.10. Statistics

The results are shown as means ± standard deviation. Statistical analyses were performed with SPSS 10.01 (SPSS Inc., Chicago, IL, USA). Because of the relatively small group sizes, non-parametric tests were used. Differences between the four treatment groups (e.g., bone formation or amount of vessels) were analyzed with the Kruskal-Wallis test for independent samples. If only two groups were compared, the Mann-Whitney test was used. The significance level was set at p ≤ 0.05. 

## 3. Results

### 3.1. VEGF Transfected Cells Led to a More Intense Angiogenesis and Vascularization than BMSCs Alone

**Figure 3:**
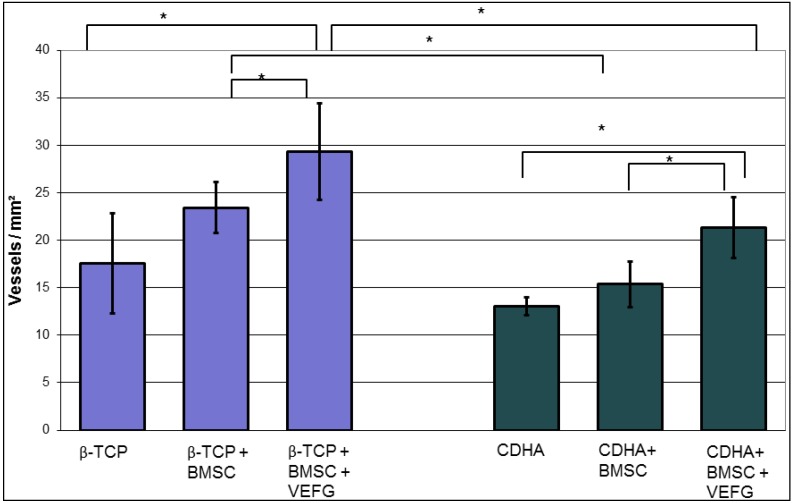
The frequency of the vessels in 6 standardized ROIs was not significantly increased by BMSCs. A significant increase was achieved by transfecting BMSCs with phVEGF_165_. In β-TCP, the vascularization was higher than in CDHA, this effect was seen before and after transfection with VEGF. (* *p* < 0.05)

Both unloaded bone substitutes showed only minor vascular ingrowth after 16 weeks, which was restricted to the periphery of the defect. There was a trend to more vascularization in the β-TCP samples that did not reach significance. Loading of the carriers with BMSCs did not lead to a significant increase of vascularization as compared to the unloaded controls. However, β-TCP loaded with BMSCs had more vascular ingrowths than CDHA loaded with BMSCs (*p* = 0.004 Mann-Whitney test). By transfecting BMSC with phVEGF_165_, angiogenesis and vascular ingrowth could be enhanced significantly on both carriers (*p* = 0.026 on β-TCP and *p* = 0.015 on CDHA, Mann-Whitney test). The combination of β-TCP with VEGF transfected cells led to the highest grade of vascularization compared to all other groups (*p* = 0.006, Kruskal-Wallis test) (Figure 3). 

### 3.2. Osteogenesis was Influenced by BMSCs and VEGF

Sequential radiographs which were taken every 4 weeks proved that none of the scaffolds dislocated or broke. Analogous to previous works [10,11], a semiquantitative evaluation of the radiographs was done. This was difficult as the carriers were not totally resorbed and denser than regular bone (Table 1). Unloaded carriers barely led to osteogenesis. Loading of both scaffolds with BMSCs increased the ossification rate. Transfection with VEGF further increased the ossification rate on β-TCP while it had a detrimental effect on CDHA. VEGF led to a higher resorption rate of CDHA after 16 weeks, but not to subsequent substitution by novel bone.

**Table 1:** Number of animals with a particular percentage of bone bridging in the radiographic evaluation

Implant	After 8 weeks				After 16 weeks			
	none	<25%	25–75%	>75%	none	<25%	25–75%	>75%
β-TCP	5	1	0	0	4	2	0	0
β-TCP + BMSC	2	3	1	0	2	0	4	0
β-TCP + BMSC+VEGF	0	3	3	0	0	0	4	2
CDHA	4	2	0	0	4	2	0	0
CDHA + BMSC	3	3	0	0	2	1	3	0
CDHA + BMSC + VEGF	3	3	0	0	3	2	1	0

### 3.3. Volume of Newly-Formed Bone Measured by µCT

**Figure 4:**
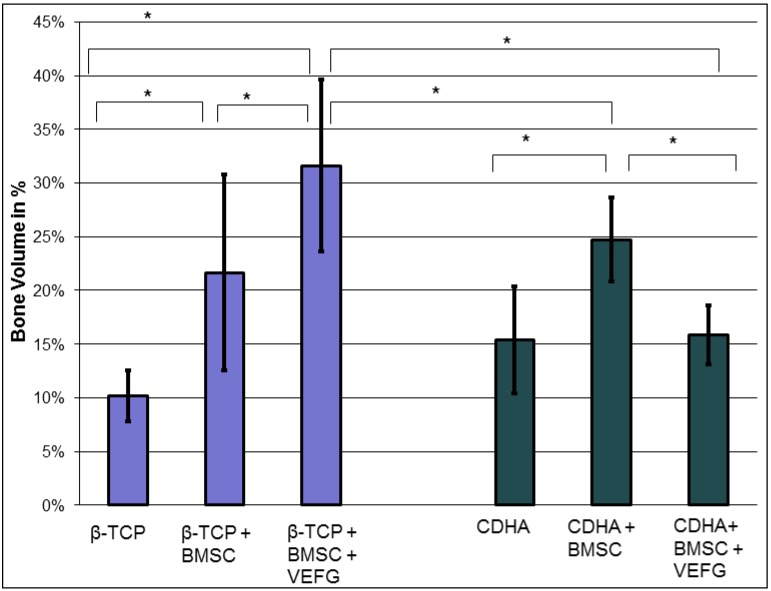
The volume of newly-formed bone was measured after retrieval of the samples in week 16. It depended on the carriers as well as on the application of BMSCs and VEGF transfected BMSCs. While the addition of BMSCs increased osteogenesis with both carriers, loading with VEGF transfected cells led to higher bone formation on β-TCP while it was inferior on CDHA. (* *p* < 0.05).

Multiplanar reconstructions of µCT scans confirmed the divergent influence of VEGF transfection of BMSCs on the volume of newly-formed bone in the two scaffolds (Figure 4). Similar results were found after 16 weeks in both types of scaffolds either unloaded or loaded with BMSCs (*p* = 0.240; Mann-Whitney Test). In both groups, BMSCs led to a significant increase of bone formation (*p* < 0.001; Mann-Whitney Test). There was no significant difference between CDHA with BMSCs and β-TCP with BMSC (*p* = 0.52; Mann-Whitney Test). However, a significant difference was found after transfection of the BMSCs with phVEGF_165_ (*p* = 0.005). While significantly more bone was found in the β-TCP group, a diminished bone volume was seen in the CDHA group. Hence, there was now a difference between the two treatment groups that was not found before loading with VEGF transfected cells (*p* = 0.004, Mann-Whitney Test), indicating that the effect of VEGF was influenced by the carrier. 

### 3.4. Resorption of the Carrier was Enhanced by VEGF

**Figure 5:**
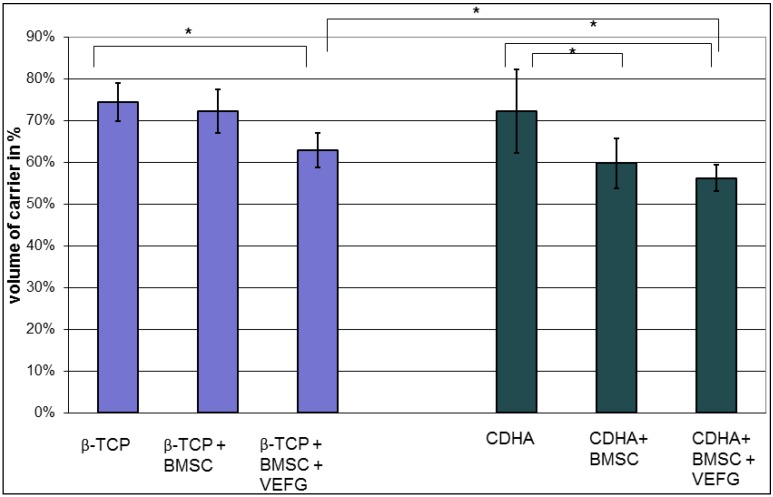
The volume of the scaffold was measured after samples were retrieved in week 16. VEGF transfected cells enhanced the degradation of both carriers. While the degradation of unloaded scaffolds was similar, BMSCs and VEGF transfected cells caused a stronger degradation of CDHA than of β-TCP. (* *p* < 0.05).

As both carriers were not totally degraded and remodeled, it was possible to determine the volume of the remaining scaffold after 16 weeks (Figure 5). While the remodeling of the unloaded carriers did not differ between both groups, BMSCs enhanced degradation of the carriers on CDHA only (*p* = 0.024; Mann-Whitney Test). The addition of transfected BMSC finally led to a significant degradation of β-TCP (*p* = 0.016; Mann-Whitney Test) which was, however, lower than on CDHA with transfected cells. Thus, there was a significant difference of the effect of VEGF transfected BMSC on the two carriers (*p* = 0.044, Mann-Whitney Test). 

## 4. Discussion

Our data demonstrate that the effect of VEGF transfected cells highly depends on the carriers they are combined with. While phVEGF_165_ transfected BMSCs boosted osteogenesis in the β-TCP group, even less bone than with regular BMSCs was found in the corresponding CDHA group. This effect was only seen after transfection of the cells with VEGF, indicating that the combination of VEGF and the carrier was responsible for the diminished osteogenesis. Without transfection of VEGF vessel formation, osteogenesis and scaffold degradation were similar when CDHA and β–TCP-groups were compared with or without BMSCs.

With both carriers, only minor osteogenesis was detected within the scaffold, if they were implanted without cells. Only minor bone grew inside the scaffold without relevant remodeling or resorption of the scaffold. By adding BMSCs without VEGF to the scaffolds, osteogenesis was enhanced significantly in both carriers, but significant degradation of the scaffold was seen only in the CDHA but not in the TCP group. Vessel density within the bone substitutes was similarly influenced in both groups: In both groups more vessels were found with BMSCs but significance was reached only with phVEGF_165_ transfected BMSCs. In the CDHA group, angiogenesis was also increased by VEGF, but not to the same extend as in the β-TCP group. 

In summary, VEGF had a positive effect on the vascularization of both carriers, but osteogenesis was only promoted in the β-TCP group. In combination with CDHA as a carrier, osteogenesis with phVEGF_165_ transfected BMSCs was smaller than with BMSCs alone. Therefore the combination of CDHA with phVEGF_165_ transfected BMSCs had the highest rate of scaffold degradation. It is possible that the higher effect on scaffold degradation inhibited simultaneous bone formation. The slower degradation of β-TCP seemed to be favorable for osteogenesis. 

We used the same cell line on both materials, pHVEGF165 is molecularly regulated, and the scaffolds are all implanted in the same type of animal, so the process of vascular formation and bone formation could logically be identical. The only thing that may change is the availability of molecular regulators of phVEGF expression due to porosity differences, inhibition of vessel formation due to surface breakdown or other properties of the scaffold, like the microenvironment (e.g., pH) introduced by its degradation.

We conclude that bone healing is influenced not only by the cells seeded on a scaffold or the addition of a growth factor, but the scaffold itself is a very important component. β-TCP and CDHA are both synthetic, resorbable scaffolds that are produced in a similar emulsion process resulting in similar micro and macropores [14]. CDHA is not sintered and has a high surface that is similar to natural bone. However, CDHA is more brittle than β-TCP, which can enhance degradation of the scaffold. The restricted osteogenesis, in combination with VEGF-transfected BMSC, might be caused by alteration of the biologic microenvironment during rapid degradation of bone substitutes [19,22]. Furthermore, fragmentation may cause instability and impair conductivity which may retard bone fusion [23]. Our data support the idea that the slower resorption of the β-TCP is favorable in combination with VEGF transfected cells. Vascularization, resorption kinetics and osteogenesis have to be in equilibrium to guarantee bone remodeling [1,17,24]. 

Consequently, the ideal bone substitute degrades at the same speed as it is replaced by bone, which preserves the stability of the construct [22]. As most bone substitutes degrade very slowly we were looking for a way to promote bone turnover. VEGF is known to promote bone remodeling by stimulating chemotaxis and activity of osteoclasts and osteoblasts, cartilage remodeling and enchondral ossification [25]. Our thesis was that it should help to remodel ceramic bone substitutes into native bone. Previous studies proved its positive effect on bone turnover and vascularization of a coralline bone substitute, but osteogenesis was impaired when the carrier degraded rapidly [11].

Only few studies compared different carriers for growth factors. Alam *et al*. [26] investigated pellet-shaped implants prepared from biphasic calcium phosphate (BCP) ceramics with different ratios of hydroxyapatite (HAP) to β-TCP impregnated with different doses of rhBMP-2 to evaluate these ceramics as bone substitutes. They found that a ratio of 25% HAP-75% β-TCP produced favorable conditions for rhBMP-2-induced bone formation compared to pure HAP. The difference was explained with the rougher surface of the β-TCP, but might as well be promoted by a higher degree of degradation [22].

Another publication by the same group [27] in which the pellets were implanted into a subcutaneous pocket instead of the cranium produced conflicting results and a higher degree of hydroxyapatite was recommended. The main difference between these two studies was the site of implantation. This indicates that the effect of a growth factor is not only influenced by the carrier but as well by the surrounding tissues and microenvironment.

Up to now we are not aware of studies investigating the influence of VEGF on different bone substitutes. Likewise, the influence of different ways of application of VEGF has been investigated in very few studies: one investigated the effect of VEGF on bone-substitutes in a setting similar to ours. Yang *et al*. [28] combined β-TCP with a VEGF-protein and found a complete bridging of a critical size defect by newly-formed bone. The administration of proteins is secure but expensive, and the release kinetics have to be considered, as VEGF needs a constant level over several days [25]. Because VEGF is a very unstable protein with a short half-life *in vivo* and very costly to produce, we chose an actively expressing transgene rather than single or multiple boluses of recombinant protein [10]. Gene therapy has the potential to maintain an optimal dose and local concentration over a period of time, thus utilizing its advantages of minimal side effects and long term efficiency [29]. 

We believe that more attention will be turned to the interaction of scaffold, cells, the combination of different growth factors and the surrounding tissues in the future. A setup that might work well on a long bone defect [30] might not work well in the intervertebral space [31] or in a non-union [2]. With growing knowledge, growth factors will be administered on distinct solid or moldable carriers and combined with distinct cells or other growth factors, depending on the site of implantation.

## 5. Conclusions

VEGF transfected BMSCs are capable of promoting angiogenesis and degradation of bone substitutes, and thus enhance remodeling and ossification of bone substitutes. The effect of VEGF depends not only on the way of administration but to a high degree on the material properties of the carrier. The combination of the growth factor VEGF, the osteogenetic cells and the matrix must be seen as entity. Up to now, too little respect was paid to the role of the bone carrier in this functional unit. This study shows its decisive influence on the capability of VEGF in bone formation.
